# How Do Escape Distance Behavior of Broiler Chickens Change in Response to a Mobile Robot Moving at Two Different Speeds?

**DOI:** 10.3390/ani14071014

**Published:** 2024-03-27

**Authors:** Glauber da Rocha Balthazar, Robson Mateus Freitas Silveira, Iran José Oliveira da Silva

**Affiliations:** 1 Ambience Research Center, Department of Biosystems Engineering, Luiz de Queiroz College of Agriculture, University of São Paulo (ESALQ/USP), Piracicaba 13418-900, Brazil; robsonsilveira@usp.br (R.M.F.S.); iranoliveira@usp.br (I.J.O.d.S.); 2 Analysis and Development Department, Federal Institute of Education, Science and Technology of São Paulo (IFSP), R. Heitor Lacerda Guedes, 1000-Cidade Satélite Íris, Campinas 13059-581, Brazil

**Keywords:** poultry behavior in response to robots, robot in poultry farm, robot movement speed in aviary, poultry escape distance in the presence of robots

## Abstract

**Simple Summary:**

The adoption of information technologies in animal farming has been growing significantly. Nevertheless, many of these technologies are still relatively new, and there is limited research on how they impact animals’ well-being. For instance, the use of robots might influence how animals perceive their habitat, potentially viewing them as intrusive elements, similar to predators or threats. Such perception can induce stress among animals, directly impacting their productivity. In broiler chickens, the perception of threat is marked by their distance and speed of escape in response to unfamiliar stimuli, such as the introduction of a robot. This study examined the escape distance undertaken by broiler chickens in the presence of a robot. Two different treatments, involving varying speeds of robot movement, were analyzed. The first treatment (lower speed of movement of the robot) determined a shorter escape distance but a greater number of collisions between robot and animal; the second (higher speed) determined greater escape distance but with fewer collisions. These results were compared with existing studies, and it was confirmed that the escape distance is related to the robot’s speed of movement, but it was also demonstrated that this relationship is inverse when analyzing the number of collisions that the robot causes to animals.

**Abstract:**

In poultry farming, robots are considered by birds as intruder elements to their environment, because animals escape due to their movement. Their escape is measured using the escape distance (ED) technique. This study analyzes the behavior of animals in relation to their ED through the use of a robot with two speeds: 12 rpm and 26 rpm. The objective is to understand whether the speeds cause variations in ED and their implications for animal stress. A broiler breeding cycle was analyzed (six weeks) through the introduction of the robot weekly. ED analyses were carried out on static images generated from footage of the robot running. The results indicate higher escape distance rates (*p* < 0.05) peaking midway through the production cycle, notably in the third week. Conversely, the final weeks saw the lowest ED, with the most significant reduction occurring in the last week. This pattern indicates a gradual escalation of ED up to the fourth week, followed by a subsequent decline. Despite RPM12 having shown low ED results, it did not show enough ED to move the animals away from their path of travel, causing bumps and collisions. RPM26 showed higher ED in all breeding phases, but showed ED with no bumps and collisions.

## 1. Introduction

Modern poultry farming has benefited from the use of various digital technologies which improve animal production, due to technologies associated with precision livestock farming [1,2]. These technologies positively influence both daily management and the obtaining of productive data which inform statistical models and, consequently, may point out production bottlenecks in breeding systems [3]. Furthermore, poultry production systems have undergone profound transformations in recent decades due to technological innovations promoted by precision livestock farming due to the benefits produced by their economic aspects. The adoption of technologies such as big data, artificial intelligence (AI), computer vision, and robotics is driven by crucial factors. These include the ability to identify and reduce losses, conduct environmental and behavioral analyses, and monitor animal welfare. Together, these aspects facilitate a deeper understanding of and solutions for productivity bottlenecks [4]. This approach enables the bridging of production gaps by integrating electronic devices with technologies that create analytical and predictive models. These models offer insights into aspects of animal production that are otherwise complex and challenging to comprehend [5]. In relation to robotics and artificial intelligence, there are no established legal mechanisms to enforce legislation that defines boundaries or regulations for the deployment of these technologies, despite their considerable potential to influence human employment. For instance, robotics can be deployed to relieve humans from working in unhealthy conditions, sparing them from tasks that may harm their well-being. However, when applied to managerial roles, this integration can lead to job displacement, raising concerns about increased unemployment [6,7,8]. Recent studies highlight a variety of robots equipped to perform tasks such as collecting eggs from aviary floors, turning over litter, sanitizing environments, and encouraging bird movement [9,10,11]. In terms of monitoring, robots outfitted with high-precision environmental sensors are capable of assessing thermal and atmospheric conditions crucial for the physiological development and welfare of animals.

Among these technologies, robotics has emerged as one of the most promising in modern poultry farming, with innovations like the Poultry Hawk, Octopus and Tibot robots [12,13]. The use of robots in poultry houses has several functionalities: collecting eggs, turning or sanitizing the litter, and collecting environmental data [14]. However, these robots may be perceived by the birds as invasive, which may lead to the birds responding with fear and escape behaviors [15]. Therefore, adopting robots in aviaries is an activity that must be carried out with great caution, and must be based on aspects which can directly affect the behavior of birds, and consequently, their well-being. The insertion of robots in poultry houses can have negative effects on the production system from the point of view of bird welfare, which has been the subject of several studies [16,17,18,19,20]. These studies analyzed the escape distance (ED) and escape speed of birds in relation to the speed of movement of robots in aviaries, with the aim of understanding how to adapt robotic technology associated with the well-being of animals, aiming to prevent a situation of fear and agitation for the animals.

High robot displacement speeds are the main influencers of ED in association with the different ages of the animals (breeding weeks); however, the lack of a greater amount of research on robotics in poultry farming limits the poultry industry’s reliance on this technology [17,18]. The hypothesis of this work is that the different movement speeds of a robot (measured through the rotations per minute [RPM] of its motorization) influence the escape distance directly, maintaining the common behavior of animals throughout the breeding cycle. For this hypothesis to occur, the habituation of the birds to the mobile robot factor was disregarded, as it was considered that by introducing the robot weekly, the birds would always reject the robot. It is suggested in future studies that the robot should be present daily when interacting with the animals, rather than weekly. This research introduces an innovative demonstrative study that explores the effects of varying motorization RPM speeds in a mobile robot through weekly insertions, coupled with an analysis of the animals’ behavior.

The general objective was to understand, between two RPM specifications (12 rpm and 26 rpm) of the same type of direct current (DC) motor, how broiler chickens behave in the presence of a mobile robot through ED analysis. The specific objectives were: determine the ED of broiler chickens during all production phases through the pixel distance analysis technique, evaluating how the engine speed specification affected the ED; evaluate which RPM promotes greater ED in animals and relate it to the experience obtained in moving the robot around the aviary in order to understand the animals’ behavior; and compare the reproducibility of two RPM speeds of a mobile robot in a commercial poultry farm.

## 2. Materials and Methods

### 2.1. Location, Characterization of the Study Area, and Ethical Regulations

The research was submitted, analyzed and approved by the Ethics Committee on the Use of Animals of the University of São Paulo, under protocol number 7364090322. The execution of this project was carried out in a commercial production of broiler chickens of the Cobb lineage, with a bed of wood shavings, located in a municipality in the State of São Paulo, Brazil (Latitude: −23.0068, Longitude: −46.8387, 23°0′24″ S, 46°50′19″ W), altitude 771 m and characterized as a humid subtropical climate with Köppen–Geiger climate classification: CF. The aviary used was of the negative pressure type and had dimensions of 135 × 14 × 3 m (L × W × H), composed of evaporative plates, exhaust fans, capacity for 20 thousand animals (21 thousand animals were housed at the time of the research) and had an electronic system for monitoring relative humidity and air temperature (inoBram brand, Pato Branco, Brazil, model SMAAI 3 PE) which acted automatically.

### 2.2. Robotic Prototyping

**Figure 1:**
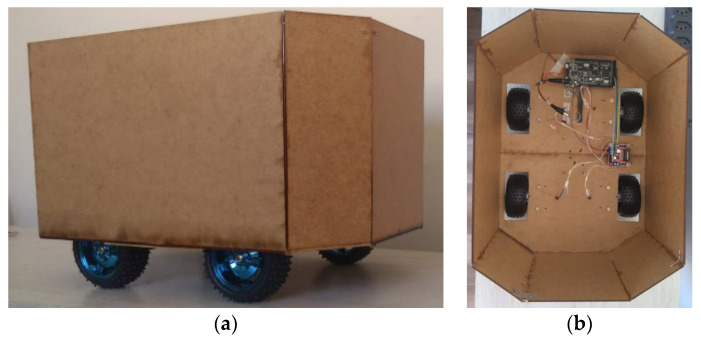
Physical prototype of the robotic structure, where: (**a**) side view; and (**b**) top internal view.

A mobile robotic chassis prototype (containing two fixed axles, four DC motors and four rubberized wheels with 75 mm diameter claws) was built following the architecture of a 4WD (4-Wheel Drive) vehicle. A single type of DC motor was established; however with different RPMs. The motors were powered by a 12 V battery and controlled by the same type of micro-controller and drive controller (H-bridge). This robot was developed to move in a uniform rectilinear way [12]. For this purpose, a single algorithm was coded in the C++ language to move the wheels. The micro-controller used was the MEGA+WiFi R3 ATmega2560+ESP8266 (Robotdyn, Hong Kong, China) and the drive controller was the HL298n (STMicroelectronics, Beijing, China). The engine model used was the DC 12v 25ga 370 Engine (XYT Motor Manufacture brand, Shenzhen Xinyongtai Motor Co., Ltd., Shenzhen, China) and the rotation specifications were 12 rpm and 26 rpm [The specifications of the engine torque model, the wheels, and the robotic chassis were the subject of a study previously carried out [21] which analyzed four different types of motors and concluded which had sufficient torque for use in commercial broiler poultry farms] (Figure 1). The dimensions of this prototype were as follows: 40 cm length, 30 cm width, 26 cm height, and 650 g in weight.

### 2.3. Data Collection

The research was carried out over 7 non-consecutive days over 6 weeks, which completes a broiler breeding cycle. For each week, two engine rotations were analyzed (treatments RPM12 and RPM26). As an insertion methodology for the robot (considered by the animals as a foreign element introduced to the aviary), its introduction was adopted by initially standing in the aviary for 30 min so that the animals could get used to its presence [17,18,19,20]. Finally, displacement analyses were performed weekly. Each weekly analysis was carried out in three executions of test displacements of the robot (test displacement1, test displacement2, and test displacement3). The experimental cycle of circulating the robot in the environment was carried out for 30 s with 5 min intervals between tests. The behaviors of 4 animals (random, totaling 144 animals in the production cycle) were analyzed weekly for each type of rotation assessed. Related studies [13,17,18,19,20] do not specify an ideal number of animals for analysis, as these studies analyzed the distance of the robotic element in relation to a group of animals. In this research, it was decided to perform analyses on four animals per experiment for standardization and behavioral assessments.

### 2.4. Escape Distance Analysis

The method used to analyze the escape distance was established from an adaptation of previous models presented in related works on the use of robotics in poultry farming [16,17,18,19,20]. The method consisted of video recording the robot’s movement through the aviary as well as the dispersion of the animals as it approached. Then, instances of the video were converted into static images (frames) in which distance analyses were carried out between pixels (conversion of pixels to centimeters) to understand the distance between the animals and the approaching robot. The pixels of this image were counted from starting to end point; then, the result found was compared with a pre-determined pixel count that represented a real scale measurement in centimeters. Finally, a measuring tape was placed on the feeder line to determine fixed markings on the robot’s trajectory, serving as a scale for the subsequent pixel counting.

**Figure 2:**
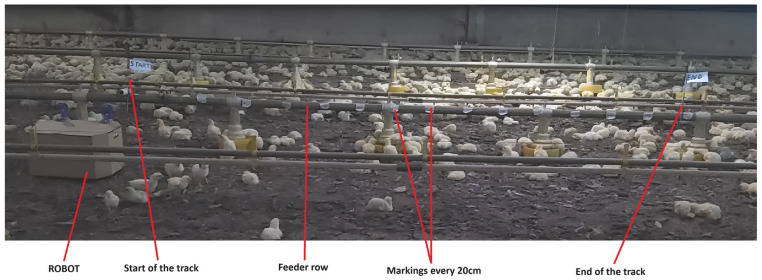
Robot runway in the commercial poultry farm.

In this way, a linear space of 3 m was delimited (called the roadway, which was positioned parallel to a row of feeders), and markings were made every 20 cm from the starting point (0 m) to the end point (3 m). This allowed the visualization of a runway on which the robot traveled a limited distance (Figure 2). Thus, the robot performed the same route in each execution, but for each execution the motors were exchanged for each respective treatment (RPM12 and RPM26). There was an interval of 30 min between the treatments for the animals to calm down and return to their natural behavior before another execution, as recommended in the literature [17,18,19,20]. The robot’s movement was carried out using an algorithm coded in C++ language that was implemented in the micro-controller present in the robotic chassis, making the robot’s movement autonomous.

The two engine models (treatments RPM12 and RPM26) were exchanged for each test performed. In order to evaluate the effects of the speed of engine rotations, the nominal speed of the engines was calculated according to the same wheel diameter of 75 mm for both. Robot displacement speeds for the two treatments are presented in Table 1, where the result of the calculation of the rotational displacement of the motor without drag (through the linear speed calculation and with the robot outside the chicken litter [22]) and carrying out the calculation of the rotational displacement of the engine with drag (by calculating the average speed with the robot acting on the chicken litter [22]).

$$v=2\times \Pi \times r\times \frac{w}{60}$$

in which v = linear speed (meters/seconds); r = radius (meters); w = angular velocity (expressed in RPM).

$$v=\frac{d}{t}$$

in which v = average speed (meters/seconds); d = distance (meters); t = time (seconds).

**Figure 3:**
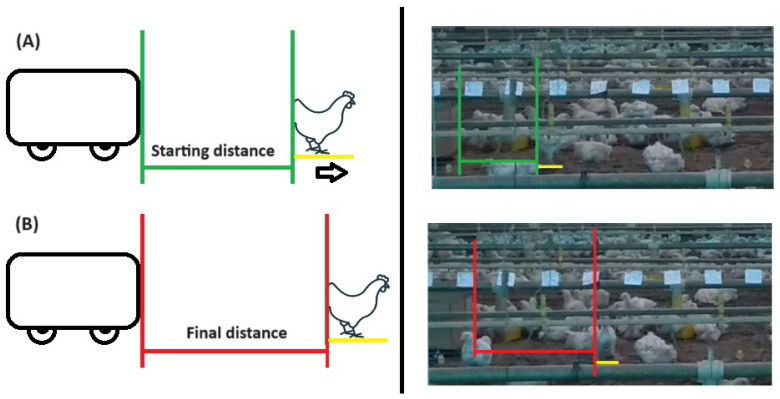
ED analysis. (**A**) Animal starts escaping (in yellow) and its distance from the robot is measured (green); (**B**) Animal finishes escaping (yellow) and its distance to the robot is measured.

All tests were filmed with a smartphone camera (High Definition 1280 × 720 pixels), Android 8.1 operating system, Asus brand (Taipei, Taiwan), Zen Phone model, which remained fixed on a pedestal at a fixed point 4 m away from the track. The ED analysis was performed by generating two static images, one capturing the starting point and the other depicting the end point of the animal’s movement. The starting point was determined by the exact moment in which the animal felt uncomfortable (animal fled in the opposite direction of the robot) with the robot’s movement and escaped (the distance from the robot to the animal was noted—Initial Distance); the final point was when the animal ended its escape by remaining still or carrying out some behavioral activity common to its species (eating, drinking water, stretching its wings and feet, cleaning feathers, lying down, etc.) without showing discomfort with the presence of the robot that continued moving (noting the distance from the robot to the animal—Final Distance) (Figure 3). The Initial Distance from the starting point was then subtracted from the Final Distance from the end point in order to determine the value in cm of escape performed by the animal. Only the escape displacements of the animals in the front part of the robot were considered, and in parallel, the fixed markings (scale) on the feeder line displacements of the animals which were perpendicular to the feeder line were disregarded, as there was no way to calculate the distance of pixels due to the fact that this model only allowed for analyzing pixel values horizontal to the camera positioning.

From the recorded markings, the distance was determined by counting the pixels between the robot and the animal (Figure 3 in green and red). In total, 288 images were evaluated, 48 images in each week of bird rearing (24 for RPM12 and 24 for RPM26). A total of 144 animals were observed in all images and it is not possible to specify whether there were repeated animals in this quantity, as the animals were analyzed randomly according to their escape behavior. The metric tool used for analyzing the distance between pixels was the free software JRuler [Available at: https://jruler.software.informer.com/3.1/ (accessed on 15 November 2023)] in version 3.1. Finally, the distance from the robot to the animal at the starting point (Figure 3 in green) was added to the distance from the robot to the animal at the end point (Figure 3 in red).

### 2.5. Statistical Analysis

The statistical analysis was divided into three stages. All analyses were performed in SPSS^®^, version 25 [23].

Step 1: Data engineering techniques were used to carry out a complete and robust analysis of the data, including the identification and treatment of outliers, aiming to ensure the integrity and reliability of the data. Subsequently, the normality of the residuals was verified using the Shapiro–Wilk test (*p* > 0.05) [24] and homoscedasticity using the Levene test [17] (*p* > 0.05). Analysis of data correlation using the Pearson test (*p* > 0.05) (adopting the “high” standard above 0.8 and significance of 95% [25]) made it possible to understand how the two treatments (RPM12 and RPM26) were related and whether there was correspondence with the literature analysis.

Step 2: The data were subjected to analysis of variance (ANOVA) to assess weeks, type of rotation and interaction effects. Averages were compared using the Tukey test (5% probability of error). The general linear model [14] used in the analysis is presented:

Yijk = µ + Si + Rj + (Si × Rj) + eijk

in which Yijk = responses of the set of dependent variables; µ = overall average; Si = effect of the i-th week (i = 1, 2… 6); Rj = effect of the j-th type of rotation (j = RPM12 and RPM26); Si × Rj = effect of interactions of number of weeks and rotations; eijk = random error.

Step 3: The intra-class correlation analysis between the two types of rotation allowed for evaluating the reliability (reproducibility) between treatments. The classification of Intraclass Correlation Coefficient (ICC) values was based on [26,27]: from 1.00 to 0.81 (excellent reproducibility); from 0.80 to 0.61 (very good); from 0.60 to 0.41 (good); from 0.40 to 0.21 (reasonable); and from 0.20 to 0.00 (poor).

### 2.6. Execution Method

The research was carried out on 4, 11, 18, 25, 32 and 39 covering six weeks (42 days) of a rearing cycle of broiler chickens. For each week, two engine rotations were analyzed (treatments RPM12 and RPM26). Each weekly analysis was carried out in three executions of test displacements of the robot (test displacement1, test displacement2, and test displacement3) and the behaviors of 4 animals (random, totaling 144 animals over the six weeks) performing escape (ED1, ED2, ED3 and ED4).

## 3. Results and Discussion

Table 2 presents the descriptive measures of the research over the six weeks and presents the averages and standard deviation.

**Figure 4:**
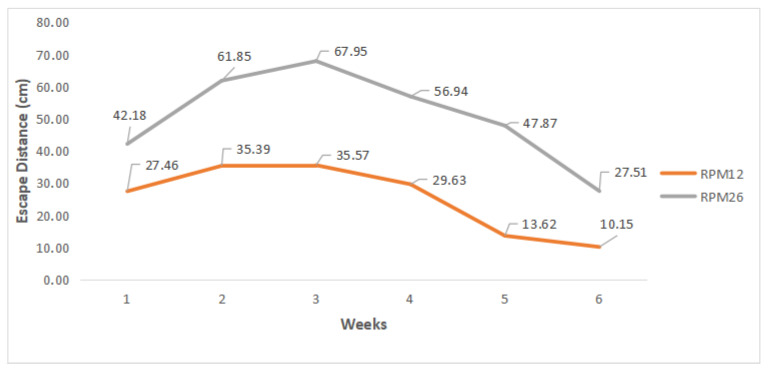
Average ED values presented for the treatments.

Figure 4 shows the evolution of ED over the six weeks of breeding for each treatment (motor rotation).

It must be considered that for the RPM12 treatment, the robotic prototype moved at a speed of 0.04 m/s, whereas in the RPM26 treatment, the speed was 0.07 m/s. This distinction is crucial for understanding why the average escape distance (ED) values at 12 rpm were lower than those observed at 26 rpm (Figure 4). The average ED value for RPM12 was 25.30 cm with a standard deviation of 14.43 cm, and for RPM26 the average value was 50.71 cm with a standard deviation of 20.14 cm. The smallest ED for RPM12 was 3.76 cm and the largest was 67.85 cm, and for RPM26 the smallest was 15.46 cm and the largest was 95.03 cm. The data showed normality with the result of the Shapiro–Wilk test *p*-value > 0.05 (Table 3) and Levene *p*-value > 0.05.

**Figure 5:**
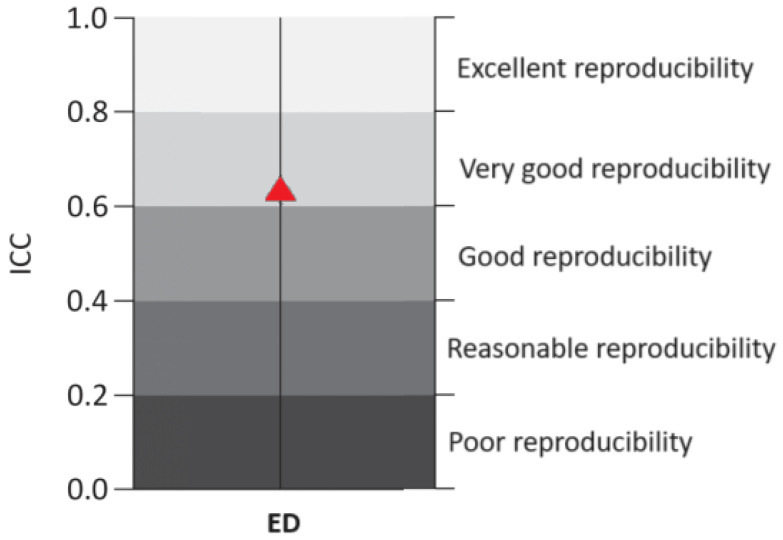
Intra-class correlation coefficient for ED of broiler chickens in commercial poultry farms. Red triangle determines the point that the equation determined on the graph indicating the degree of reproducibility.

The result of the correlation analysis between the ED of the two treatments was r = 0.83. This demonstrated that there was a strong, significant and positive correlation between the rotations evaluated. The two rotations, despite having different escape distances, displayed similar changes in escape distance behaviors. The same result was found through intra-class correlation analysis (Figure 5) between the RPM12 and RPM26 treatments to determine ED; this presented a result *p* = 0.621 (*p* < 0.001) which determined very good reproducibility (>0.60 ICC < 0.80) (reproducibility describes the degree of relationship between the two treatments, thus confirming the degree of correlation between them).

There was no interaction effect between the type of rotation and the weeks (*p* > 0.05). Therefore, the effects are discussed in isolation. A higher rate of escape distance (*p* < 0.05) was observed in the second, third and fourth weeks with greater significance in the third; on the other hand, a lower rate of escape distance was observed from the fifth week onwards. It is understood that there was an increasing movement in ED until the 3rd week of rearing (21 days) and subsequently, there was a decrease in ED. This response pattern corroborates [19,20] as both observed that, over time, EDs tend to decrease with the presence of the robot (authors state that animals tend to get used to the presence of objects inside the aviary, and the robot is no different). In agreement, [17,18] add that the age of the animals is directly related to ED. Younger animals (more active) tend to have greater ED with the presence of the robot, whereas older animals (less active) tend to have lower ED.

This response pattern is possibly also explained by the association with the size and weight of the birds: young and lighter animals are more active, whereas adult and heavier animals are less active. During the research on the RPM12 treatment, cases of very low ED (less than 10 cm) were found, specifically in the last two weeks (Table 2). In the field, this represented a very close proximity of the robot to the animals, causing it to bump into the animals and forcing them to stand up to move out of the way. This is related to the fact that broiler chickens at advanced ages present greater difficulty of movement due to their body weight and also the high density of animals per square meter [20]. Nevertheless, the RPM26 treatment maintained a safe distance between the robot and the animals across all weeks of rearing, preventing contact due to its higher escape distances (EDs). This was especially evident in the first three weeks, where exceptionally high ED values indicated a pronounced avoidance behavior, likely stemming from the animals’ excessive fear of the robot, which was perceived as a threat or predator [9,10] (Table 4).

The RPM12 treatment displayed the lowest escape distance (ED) values (*p* < 0.05; Table 5), confirming the hypothesis that a robot’s movement speed directly influences ED (the higher the speed, the higher the ED). Nonetheless, the animals exhibited varied behaviors in response to the robot’s presence within the aviary, indicating that while movement speed is a critical factor, other variables also play a significant role in shaping animal behavior in such contexts. The RPM12 treatment showed lower ED in all weeks when compared to the RPM26 treatment (Figure 4). This led to a greater adaptability of the animals to the robot’s presence, without causing panic-induced dispersion. Panic is identifiable by the rapid escape of one or more animals, which can spread fear to others previously undisturbed by the robot, potentially causing them to clump together. However, this low ED was not enough to keep the animals away from the robot’s movement in the fifth and, mainly, in the sixth week, which led to several collisions between the robot and the animals. The RPM26 treatment resulted in behaviors that suggested a heightened level of fear among the animals across all rearing weeks due to the constantly high ED values presented. Nevertheless, during the final weeks, when the animals tend to present more mobility issues, this displacement speed was enough to move the animals away from the path of the robot, avoiding collisions.

The behavior analyzed for the RPM12 treatment demonstrated that this speed is unable to disperse the animals in the final weeks, causing collisions. These collisions can cause injuries to animals and, consequently, damage to their bodies. However, it is suggested to analyze the RPM26 treatment with daily insertions and not just weekly in order to check if the ED decreases, as a new question arises: does the ED decrease with daily insertions of the robot throughout the breeding cycle instead of just weekly insertions due to the adaptability of the animals as suggested in [17,18]?

## 4. Limitations

The fact that the animals walked very slowly in the final weeks demonstrates that a greater speed of a robot does not influence greater ED in these weeks. However, it is suggested that further research should focus on other rotational speeds (above 26 rpm) to analyze this hypothesis in more detail. Furthermore, another important aspect to be considered is that at first, the mobile robot is a novelty in the production environment. It is believed that if the robot were used daily, the ED would probably be smaller, as the robot would not be considered an intrusive element and the behavior learned by the birds would maintain a normal escape distance. Finally, it is recommended and encouraged to conduct studies evaluating prototypes with varying speeds tailored for each phase of animal husbandry. This approach aims to explore the relationship between age (animal weight), escape distance (ED), and speed (RPM).

## 5. Implications

This work is part of a research which aims to build a robot to work in commercial broiler poultry farms, called RobôFrango. The results of the 26 rpm rotation were sufficient to determine an engine specification that can operate in an aviary, and to predict the impact of an intruding element on the animals’ behavior within their habitat.

## 6. Conclusions

The behavior of the animals did not change due to the insertion of the robot as the animals showed the expected behavior (they begin their life cycle being very active [initial weeks: 1, 2 and 3] and tend to decrease their movement at the end [final weeks: 4, 5 and 6]). Each treatment presented different EDs weekly, but both were correlated with each other and with the natural movement behavior of the animals. It is concluded that the highest speed treatment (RPM26) does not cause collisions in the final weeks but presents a higher ED when compared to the other treatment (RPM12) which presents a lower ED throughout the cycle but in the final weeks collides with the animals and can cause injuries.

## Figures and Tables

**Table 1:** Average values of the displacement speed of the robotic structure in a commercial poultry farm.

Treatment	Linear Speed	Average Speed	
	Travel Speed (m/s)	Travel Speed (m/s)	Distance Moved (cm)
RPM12	0.08	0.04	95 ± 0.05
RPM26	0.18	0.07	110 ± 0.09

**Table 2:** Values found for the escape distance of the animals per treatment.

Week	Treatment	Verified Values (cm)					Average	Standard Deviation
		Executions	ED1	ED2	ED3	ED4		
1	RPM12	DT1	12.08	16.17	10.54	10.54	27.46	14.13
		DT2	27.13	45.01	45.01	47.38		
		DT3	20.91	23.87	29.79	41.11		
	RPM26	DT1	15.46	26.18	54.37	64.26	42.17	16.80
		DT2	67.34	42.35	48.09	51.41		
		DT3	41.16	16.47	33.52	45.49		
	Totals:						34.81	16.94
2	RPM12	DT1	22.11	24.58	28.34	21.55	35.38	13.47
		DT2	38.39	29.86	22.34	67.86		
		DT3	41.65	43.72	41.81	42.43		
	RPM26	DT1	48.55	60.62	57.59	49.28	61.85	15.65
		DT2	84.81	47.71	48.72	73.64		
		DT3	90.31	46.02	76.95	58.04		
	Totals:						48.62	19.66
3	RPM12	DT1	15.39	42.16	42.48	22.03	35.57	9
		DT2	42.16	47.92	34.38	35.85		
		DT3	37.37	34.38	38.24	34.49		
	RPM26	DT1	22.14	70.12	64.46	86.22	67.94	18.89
		DT2	63.32	62.77	95.03	91.55		
		DT3	58.64	62.83	65.82	72.46		
	Totals:						51.75	21.97
4	RPM12	DT1	25.94	57.47	32.44	29.26	29.63	12.06
		DT2	19.39	37.13	16.44	38.49		
		DT3	22.53	35.18	29.27	12.06		
	RPM26	DT1	28.62	48.73	48.06	84.93	56.93	17.64
		DT2	81.69	35.46	56.93	67.81		
		DT3	62.14	50.77	44.45	73.69		
	Totals:						43.28	20.32
5	RPM12	DT1	11.53	21.29	7.76	5.41	13.62	7.78
		DT2	23.20	6.70	18.66	8.87		
		DT3	8.75	24.76	22.37	4.14		
	RPM26	DT1	38.04	28.88	34.39	49.63	47.86	14.57
		DT2	63.44	35.63	60.42	65.10		
		DT3	57.57	35.31	69.48	36.54		
	Totals:						30.74	20.89
6	RPM12	DT1	18.36	17.10	15.19	12.02	10.14	5.09
		DT2	11.86	12.57	6.50	9.13		
		DT3	5.66	3.76	5.03	4.60		
	RPM26	DT1	20.55	24.59	34.86	31.99	27.51	5.87
		DT2	38.38	26.13	32.36	24.66		
		DT3	29.02	19.34	25.90	22.40		
	Total:						18.83	10.37
Total RPM12:							25.30	14.43
Total RPM26:							50.71	20.14
Grand total:							38.01	21.62

ED: Escape distance; DT: Test displacement.

**Table 3:** Normality test.

	Shapiro–Wilk	Levene
	*p*-Value	*p*-Value
Average ED	0.231	0.087

**Table 4:** Escape distance of birds according to weeks of breeding.

Week	Animals	Average	Standard Error
1st week	24	34.82 bc	3.46
2nd week	24	48.62 ab	4.01
3rd week	24	51.76 a	4.49
4th week	24	43.29 abc	4.15
5th week	24	30.74 cd	4.26
6th week	24	18.83 d	2.12

a–d: different lowercase letters differ by Tukey’s test with 5% probability of error.

**Table 5:** Escape distance according to engine speed.

Treatment	Animals	Average (cm)	Standard Error (cm)
RPM12	72	25.30 b	1.70
RPM26	72	50.72 a	2.37

a, b: different lowercase letters differ by Tukey’s test with 5% error probability.

## Data Availability

The datasets generated and/or analyzed during the current study are available upon request to the corresponding author. The data are not publicly available due to these are high-resolution video files that take up too much space to be available in the cloud.
